# Binocular summation in high and low contrast letter acuities

**DOI:** 10.3389/fnins.2023.1174900

**Published:** 2023-06-15

**Authors:** Deyue Yu, Emily Watson

**Affiliations:** College of Optometry, The Ohio State University, Columbus, OH, United States

**Keywords:** binocular summation, visual acuity, contrast, normal vision, letter recognition

## Abstract

Binocular summation, a well-known phenomenon in letter acuity measurement, refers to the improvement in visual performance when viewing with both eyes compared to one eye alone. The present study aims to assess the relationship in binocular summation between high and low contrast letter acuities, and examine whether baseline measure (binocular summation at either high or low contrast) is predictive of the change in binocular summation between contrast conditions. Corrected high and low contrast letter acuities were assessed monocularly and binocularly in 358 normal vision observers aged 18–37 years using Bailey-Lovie charts. All observers had high contrast acuities (both monocular and binocular) of 0.1 LogMAR or better and no known eye disease. Binocular summation was calculated as the difference in LogMAR between the better eye acuity and binocular acuity. We found that binocular summation was present at both contrast levels (0.044 ± 0.002 LogMAR for high and 0.069 ± 0.002 LogMAR for low contrast) with higher magnitude of summation at low contrast, and declined with increasing interocular difference. There was a correlation in binocular summation between high and low contrast. The difference in binocular summation between the two contrast levels was found to be correlated with the baseline measurement. Using common commercially available letter acuity charts, we replicated the findings on binocular acuity summation in normally sighted young adults for both high and low contrast letters. Our study revealed a positive relationship in binocular acuity summation between high and low contrast, and an association between a baseline measure and the change in binocular summation between contrast levels. These findings may serve as a reference in clinical practice and research when high and low contrast binocular summations are measured in assessing binocular functional vision.

## Introduction

When comparing monocular and binocular visual acuities, an observer may exhibit binocular summation, equivalence, or inhibition (Cagenello et al., 1993; Rubin et al., 2000; Azen et al., 2002; Pineles et al., 2011, 2014; Lee and Choi, 2017). Binocular summation refers to the improvement in visual performance when viewing with both eyes compared to one eye alone. Binocular inhibition is the term used most often to describe negative binocular summation, where binocular acuity is actually worse than monocular acuity. Although the precise mechanism involved in binocular inhibition is unclear, it is thought to be connected to interocular suppressive mechanisms in layer V1 of the visual cortex (Baker et al., 2007; Moradi and Heeger, 2009; Pineles et al., 2013). Binocular summation, typically calculated as the difference between the acuity of the better eye and the binocular acuity (Binocular summation = LogMAR _better monocular_–LogMAR _binocular_), is present at both high (close to 100%) and low contrast (e.g., 11, 2.5, and 1.25%), with higher magnitude at low contrast (Home, 1978; Blake et al., 1981; Pineles et al., 2011, 2014). According to the probability summation model, the superiority of binocular over monocular viewing is anticipated since presenting stimuli to two eyes concurrently doubles the likelihood of a correct response (Blake and Fox, 1973). However, many lines of evidence point to the possibility that binocular summation has a cortical basis and occurs as a result of neural summation of the signals from both eyes (Campbell and Green, 1965; Poggio and Fischer, 1977; Legge, 1984; Tarita-Nistor et al., 2006). Since deviations from the normal range of binocular summation are often linked to pathological conditions especially at low contrast, incorporating binocular summation as a metric in the assessment of binocular functional vision has been considered beneficial (Taylor et al., 1991; Pardhan, 1993; Pineles et al., 2013, 2015). In low vision services, measure of binocular summation may also help guide the selection of suitable low vision aids and rehabilitation to optimize residual vision of a patient (Tong et al., 2021).

Interocular difference in visual acuity quantifies the within-subject asymmetry of visual function between the two eyes, and have been commonly used for detecting abnormal visual function and early pathological changes. Binocular superiority occurs more frequently when the monocular acuities of the two eyes are similar. While having unequal monocular acuities does not eliminate binocular summation or warrant binocular inhibition (Rubin et al., 2000), people with larger interocular differences more likely exhibit decline in binocular summation and even binocular inhibition, especially at low contrast (Azen et al., 2002; Pineles et al., 2011). The range of interocular difference in a normal population is 0.16 LogMAR for high contrast visual acuity and 0.17 LogMAR for low contrast acuity (Wood and Bullimore, 1996). At low contrast, interocular difference shows small increase with age, possibly because letter acuity measure obtained at low contrast is more sensitive to small inter-eye differences in ocular media clarity (Wood and Bullimore, 1996). As might be expected, aging is also linked to diminished binocular summation and binocular inhibition (Pardhan, 1996, 1997; Pineles et al., 2011) in particular at low contrast (Gagnon and Kline, 2003; Pineles et al., 2011, 2014).

Bailey-Lovie visual acuity chart is widely recognized as the gold standard for its design principles and accuracy (Bailey and Lovie-Kitchin, 2013). It can be used in clinical practice as well as research for patients with normal and low vision. The chart adopts a proportionally spaced sans-serif font, a fixed number of letters in each row, a uniform logarithmic increment of letter size and spacing, and standardized scoring methods. The Bailey-Lovie chart is produced in both high and low contrast versions. While visual acuity is typically measured using high contrast letters (black letters on white background), it has been demonstrated that low contrast chart can provide further information valuable for detecting and assessing visual deficits (Brown and Lovie-Kitchin, 1989; Schneck et al., 2004; Wieder et al., 2013). Abnormal binocular summation is usually indicative of pathological condition and the measurements of binocular summation obtained at different contrast levels can convey distinct information (Taylor et al., 1991; Pardhan, 1993; Pineles et al., 2013, 2015). Therefore, assessing binocular summation using both high and low contrast acuity charts may be necessary for gaining a comprehensive picture of binocular functional vision. An unusual change of binocular summation between the two contrast levels may have implications on revealing and identifying visual impairments.

In this study, we measure binocular visual acuity summation in normally sighted young adults using common commercially available visual acuity charts (high and low contrast Bailey-Lovie charts) and assess the relationships of various acuity-based measurements. The main purpose is to investigate whether people who exhibit stronger binocular acuity summation at high contrast tend to have greater summation at low contrast, and whether baseline measure (binocular summation at either high or low contrast) is predictive of the change in binocular summation between contrast conditions.

## Methods

### Observers

A total of 358 normally sighted observers aged between 18 and 37 years were enrolled for various studies from 2013 to 2022 in the laboratory of Deyue Yu. Four of the studies were published (Husk and Yu, 2015; Shepard et al., 2019, 2021; Treleaven and Yu, 2020) and the rest were unpublished. Of the enrolled observers, 212 were female and 146 were male. All data were collected with informed written consent approved by the institutional review board of The Ohio State University. The research followed the tenets of the Declaration of Helsinki. All observers met the following inclusion criteria: adults younger than 40 years of age, having monocular and binocular high contrast acuities of 0.1 LogMAR or better (counting all correct responses), and having no known eye disease.

### Visual acuity assessments

**FIGURE 1:**
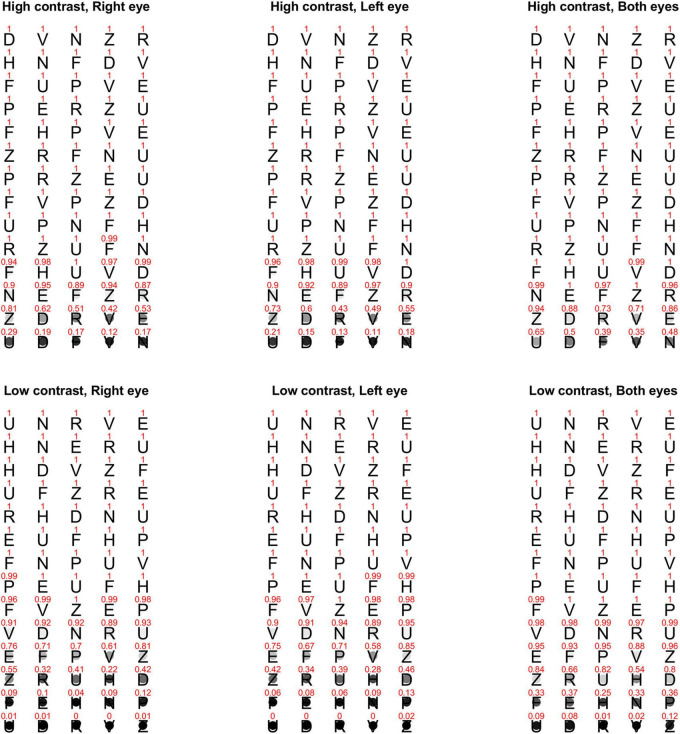
Proportion of correct responses for each letter on the high and low contrast Bailey-Lovie charts that were used for measuring monocular and binocular visual acuities at three-meter viewing distance. The top row is the high contrast chart, and the bottom row is the low contrast chart. The three columns are for right eye, left eye, and both eyes, respectively. The value above each letter stands for the proportion of correct responses across all observers. The same information is also represented with a gray circle on **(top)** of each letter with black being 0% correct and white/transparent being 100% correct. The Bailey-Lovie charts use British Standard letters, gray letters against the white background for the low contrast chart, and decreasing letter size and spacing from **(top)** to **(bottom)**. For illustrative purposes, the letters in this figure are portrayed as black letters (in Helvetica font) on white background for both contrast levels and having the same size and spacing.

High and low contrast Bailey-Lovie charts (Bailey and Lovie, 1976) were used to measure best-corrected visual acuities both monocularly and binocularly at three meters. The acuity charts were illuminated using customized lighting to provide a uniform background luminance of 110 cd/m^2^. The low contrast chart had a Weber contrast of 18%. The version of charts used for the two contrast levels are shown in Figure 1. All observers completed the tests with their habitual glasses or contact lenses if any. High contrast visual acuity was always tested before low contrast visual acuity. For each contrast level, monocular acuities were always measured first and then binocular acuity. To mimic the quick measurements recorded in clinical settings, for each contrast level, we used the same chart for all monocular and binocular measurements with reading direction reversed after each acuity measure. Right eye was tested first with observers reading from left to right, followed by left eye with right to left reading direction. Binocular measure was always obtained last with a left to right reading direction. Following the standard procedure, observers were instructed to start from the top row of each chart and progress to the smallest size that could be read. Observers were encouraged to provide their best guesses when they were uncertain, and stopped when they could no longer correctly identify additional letters. As shown in our data, the two monocular acuities were nearly identical [high contrast: *t*(357) = −1.17, *p* = 0.24; low contrast: *t*(357) = −0.78, *p* = 0.44], and the correctly recognized letters near the acuity limits were often different between the two eyes, indicating minimal gain from using the same chart repeatedly.

### Data analysis

To estimate visual acuity, we adopt the letter-by-letter scoring method (each correctly reported letter worth 0.02 LogMAR), and the most commonly used termination rule—stopping counting when three or more mistakes have been made on a five-letter row (Williams et al., 2008; Carkeet and Bailey, 2017). Lower value in LogMAR represents better visual acuity. For each contrast level, the better monocular acuity is defined as the lowest LogMAR value obtained monocularly.

Both binocular summation and interocular difference are obtained from the visual acuity scores for each of the two contrast levels. Binocular summation is computed as the difference between the better monocular acuity and the binocular acuity (LogMAR _better monocular_–LogMAR _binocular_). For instance, if binocular acuity is −0.1 LogMAR and better eye acuity is 0.0 LogMAR, the binocular summation would be 0.1 LogMAR. A positive difference (i.e., when the binocular visual acuity is better than the better monocular acuity) suggests binocular summation. A negative difference indicates binocular inhibition. Larger amplitudes represent larger summation/inhibition. Interocular difference is calculated as the difference between the better and worse monocular acuity (LogMAR _better monocular_–LogMAR _worse monocular_), with larger absolute values representing larger interocular difference.

We examined monocular and binocular visual acuities, interocular difference and binocular summation at two contrast levels, and the correlations between various measurements. Specifically, we hypothesized a positive correlation between high and low contrast for measurements including monocular acuities, binocular acuity, interocular difference, and binocular summation, and a positive correlation between monocular and binocular acuity. According to previous research, we also expected that binocular summation was negatively correlated with interocular difference and age, and that interocular difference increased with age. To evaluate the above hypotheses, one-tailed correlation tests were performed. For the rest of the analysis, two-tailed tests were used. The False discovery rate (FDR) correction was implemented to correct for multiple comparisons (Benjamini and Hochberg, 1995). A *p*-value of 0.05 or less was considered statistically significant.

A viewing distance of three meters was used in the present study. Since the standard distance for Bailey-Lovie acuity charts was six meters, an appropriate adjustment (+ 0.3 LogMAR) was made to the LogMAR score. The range of letter size was changed from 0.8 to −0.5 LogMAR at six meters to 1.1 to −0.2 LogMAR at three meters. Unfortunately, the size range after adjustment was not always sufficient to avoid truncation, that is, some observers were able to read more than two letters in the last row (according to the termination rule, they should continue reading) and might be able to read more letters if an additional row of letters with smaller size was available. As a results, we repeated the analyses after excluding these observers (a total of 156 observers left) to test if it would impact the results. Similar findings were obtained (see Supplementary Appendix A).

## Results

### Visual acuities, interocular difference, and binocular summation

Table 1 lists the monocular and binocular acuities, interocular differences and binocular summations at both contrast levels, and the differences between the two contrast levels.

**TABLE 1:** Monocular and binocular acuities, interocular differences (LogMAR _better monocular_–LogMAR _worse monocular_) and binocular summations (LogMAR _better monocular_–LogMAR _binocular_) for the two contrast levels, the differences between the two contrast levels (Mean ± SE in LogMAR), and correlations between the two contrast levels.

	High contrast	Low contrast	High–Low	*r*	One-tailed *p*
Monocular (right)	−0.065 ± 0.004	0.090 ± 0.005	−0.156 ± 0.004	0.66	<0.001
Monocular (left)	−0.062 ± 0.004	0.093 ± 0.005	−0.155 ± 0.004	0.62	<0.001
Monocular (better)	−0.084 ± 0.003	0.065 ± 0.005	−0.149 ± 0.004	0.66	<0.001
Monocular (worse)	−0.043 ± 0.003	0.118 ± 0.005	−0.161 ± 0.004	0.62	<0.001
Binocular	−0.128 ± 0.003	−0.004 ± 0.005	−0.125 ± 0.004	0.65	<0.001
Interocular difference	−0.041 ± 0.002	−0.053 ± 0.003	0.012 ± 0.003	0.37	<0.001
Binocular summation	0.044 ± 0.002	0.069 ± 0.002	−0.025 ± 0.003	0.09	0.04

The top four rows are monocular visual acuities for right, left, better seeing, and worse seeing eye, respectively.

For both monocular and binocular measurements, the high contrast acuities were always better than the low contrast acuities (more than one-row difference, *p*s < 0.001). However, interocular difference and binocular summation were both greater at low contrast than high contrast by small amount (*p*s < 0.001).

**FIGURE 2:**
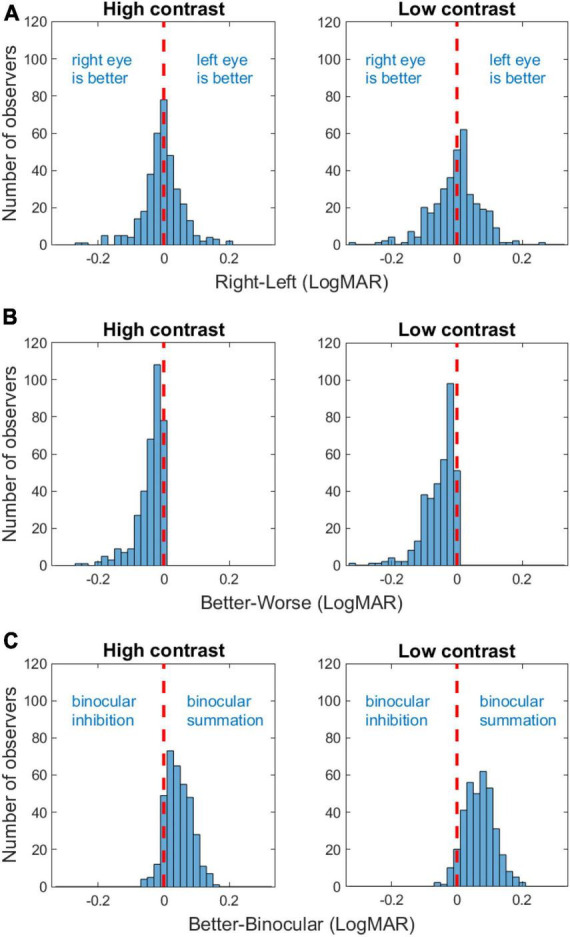
Frequency histograms of acuity difference between the right and the left eye **(A)**, interocular difference [acuity difference between the better and the worse eye; **(B)**], and binocular summation **(C)** for high and low contrasts. The red vertical dashed lines indicate zero difference.

At both contrast levels, visual acuity was similar between the right and the left eye with the difference ranging between −0.26 and 0.20 LogMAR for high contrast (mean = −0.004 LogMAR; paired *t*-test: *t*(357) = −1.17, *p* = 0.24) and −0.32 and 0.26 LogMAR for low contrast (mean = −0.003 LogMAR; paired *t*-test: *t*(357) = −0.78, *p* = 0.44; Figure 2A), indicating minimal learning effect between the two measurements. Binocular acuity was consistently better than monocular (right, left, better, and worse eye) acuities (*p*s < 0.001). Figures 2B, C showed the distributions of interocular difference and binocular summation. Interocular difference was found in 78% of the observers for high contrast (a difference of −0.26 LogMAR or less) and 86% for low contrast (−0.32 LogMAR or less; Figure 2B). The amplitude of interocular difference in the majority of our observers fell within the range of normality (Wood and Bullimore, 1996), with only a minor proportion exhibiting deviations (3% for both high and low contrast levels). Among the observers who had interocular difference at both contrast levels, 30% switched their better eye when contrast level changed. Among all the observers, 80% showed some degree of binocular summation for high contrast chart (up to 0.16 LogMAR) and 91% for low contrast chart (up to 0.20 LogMAR; Figure 2C). The rest of the observers either had no change or showed a small amount of binocular inhibition.

### Correlations

**FIGURE 3:**
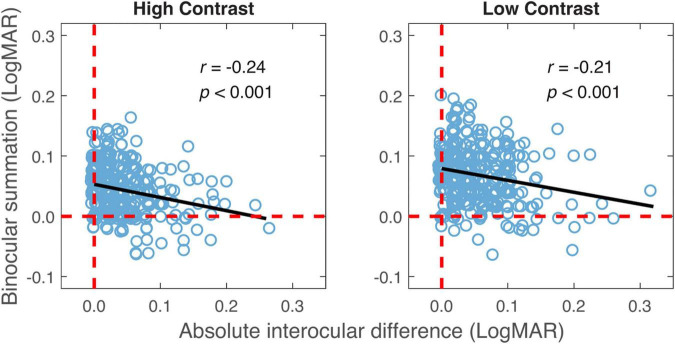
Binocular summation plotted against absolute interocular difference for high and low contrasts. Black line represents the best-fitting line to the data. Random horizontal and vertical jitters (range between –0.005 and 0.005) are added to each data point on the plot in order to separate overlapping points. The red dashed lines mark zero binocular summation or zero interocular difference.

Consistent with previous findings (Azen et al., 2002), there was a positive correlation between the better eye acuity and binocular acuity, and a lower correlation between the worse eye acuity and binocular acuity for the high contrast chart (Table 2). Similar correlations were observed for the low contrast chart as well. As shown in Table 2, significant correlation was also found between binocular summation and absolute interocular difference. The observers with larger interocular differences were more likely exhibit decline in binocular summation and even binocular inhibition (see Figure 3). In other words, binocular summation tended to be present when visual acuities between the two eyes are closely matched. As shown in Table 1, significant correlations were consistently found between the high and low contrast measures. Consistent with the finding of previous study (Pineles et al., 2014), correlation between age and binocular summation was found significant for low contrast chart only (*r* = −0.18, one-tailed *p* < 0.001). No correlation was found between age and interocular difference.

**TABLE 2:** Correlations between binocular and monocular (better or worse) acuity, between binocular summation and absolute interocular difference for the two contrast levels.

		High contrast		Low contrast	
		*r*	One-tailed *p*	*r*	One-tailed *p*
Binocular	Better	0.79	<0.001	0.87	<0.001
	Worse	0.66	<0.001	0.81	<0.001
Binocular summation	| Interocular difference|	−0.24	<0.001	−0.21	<0.001

**FIGURE 4:**
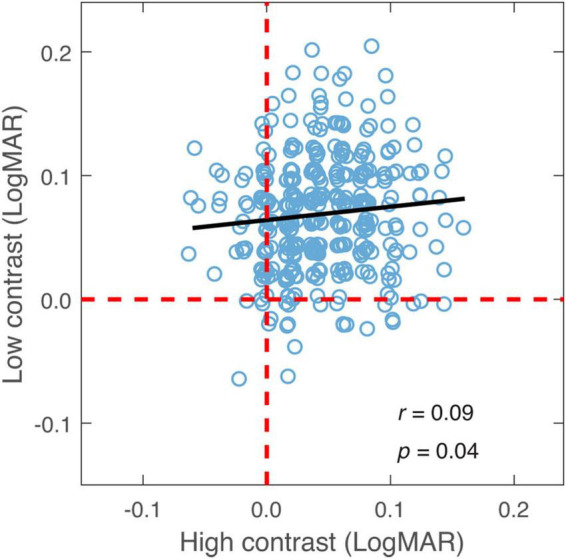
Binocular summation at low contrast plotted against binocular summation at high contrast. Black line represents the best-fitting line to the data. Random horizontal and vertical jitters (range between –0.005 and 0.005) are added to each data point on the plot in order to separate overlapping points. The red dashed lines mark zero binocular summation at low and high contrast levels.

**FIGURE 5:**
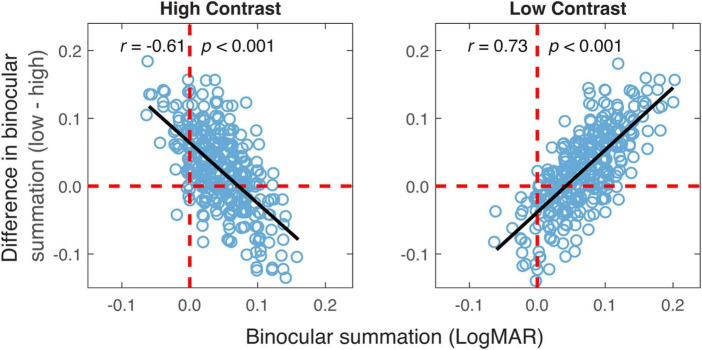
The difference in binocular summation between the two contrast levels plotted vs. binocular summation at high and low contrast levels. Black lines represent the best-fitting lines to the data. The vertical red dashed lines represent zero binocular summation at a given contrast level. The horizontal red dashed lines represent no difference in binocular summation between the two contrast levels. The data points above the dashed line are the ones having greater binocular summation for low contrast acuity.

The relationship in binocular summation between high and low contrast was examined to decide whether observers who exhibited stronger binocular summation at high contrast tended to have a greater summation at low contrast. As shown in Figure 4, binocular summation at low contrast had a positive correlation with summation at high contrast (*r* = 0.09, one-tailed *p* = 0.04). We then examined whether the change in binocular summation between two contrast levels depends on the level of baseline measurement. Here, the baseline measurement was binocular summation at either high or low contrast. We found that the change of binocular summation correlated significantly with binocular summation measured at both contrast levels (Figure 5). The correlation was negative with high-contrast binocular summation (*r* = −0.61, *p* < 0.001) and positive with low-contrast binocular summation (*r* = 0.73, *p* < 0.001), indicating a correspondence of a larger change in binocular summation with lower binocular summation at high contrast and higher binocular summation at low contrast. Due to methodological concerns of mathematical coupling (occurring when one variable contains the other) and regression to the mean, Oldham’s method was used to provide an unbiased test of the correlation between change of binocular summation and baseline measurement (Tu and Gilthorpe, 2007), and further confirmed that the effect of contrast depended on baseline (*r* = 0.14, *p* = 0.01). In short, we identified a direct relationship in binocular acuity summation between high and low contrast, and showed that baseline measure is predictive of the change in binocular summation between contrast conditions.

**FIGURE 6:**
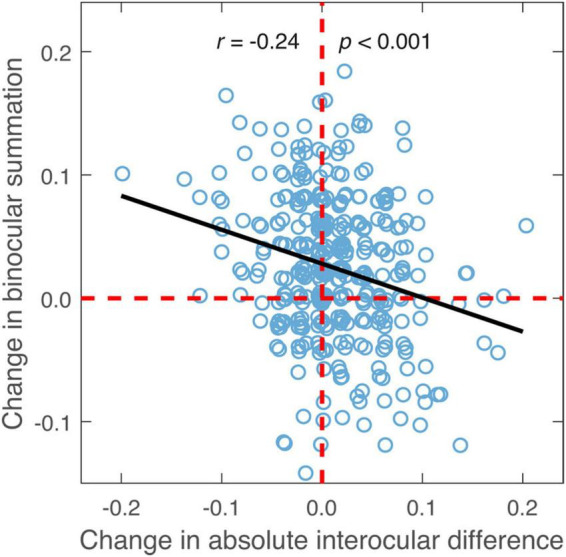
The difference in binocular summation plotted against the difference in absolute interocular difference (low contrast—high contrast; in LogMAR units). Black line represents the best-fitting line to the data. Random horizontal and vertical jitters (range between –0.005 and 0.005) are added to each data point on the plot in order to separate overlapping points. The red dashed lines mark zero differences.

The change of binocular summation between the two contrast levels was also found to correlate negatively with the change of absolute interocular difference (*r* = −0.24, *p* < 0.001; Figure 6) and age (*r* = −0.14, *p* = 0.009).

## Discussion

Using common commercially available letter acuity charts, we replicated the findings on binocular acuity summation in normally sighted young adults for both high and low contrast letters, with overall greater summation at low contrast. The amount of binocular summation in our observer group was comparable to that reported in earlier studies (Azen et al., 2002; Pineles et al., 2014). The main objective of our study was to assess the relationship in binocular summation between high and low contrast letter acuities, and examine whether baseline measure was predictive of the change in binocular summation between contrast conditions. We observed a positive correlation in binocular summation between high and low contrast indicating that observers who exhibited stronger binocular summation at high contrast tended to have a greater summation at low contrast. This relationship can be reasonably anticipated based on relevant neurophysiological findings. A recent study by Mitchell et al. (2022) revealed multiple steps of processing when examining relationship between binocular facilitation in primary visual cortex and stimulus contrast, with the initial binocular processing being more contrast-invariant and the subsequent processing being more contrast-dependent. The study showed overall greater binocular facilitation at lower contrast compared to higher contrast, which is in line with the psychophysical findings on binocular summation. The correlations between binocular summation measures that we observed here may reflect the influence of the combined common and distinct processing across contrast levels. It is possible that besides abnormal binocular summation, an unusual change of binocular summation between the two contrast levels may convey additional information useful for detecting and assessing visual deficits. While we also found that the difference in binocular summation between the two contrast levels was dependent on the baseline measurement of binocular summation, the exact relationship should be interpreted with due caution given the presence of mathematical coupling.

Considering the range of interocular difference in a normal population (Wood and Bullimore, 1996), only 3% of our observers fell outside the range. Consistent with previous findings (Blake et al., 1981; Azen et al., 2002), we found that binocular summation was more likely to be present or higher in magnitude when monocular visual acuities are closely matched. The observers with substantial interocular differences were more likely to exhibit binocular inhibition or a reduction in binocular summation. This is true for both high and low contrast acuities. Our results also revealed a negative association between the change in binocular summation and the change in absolute interocular difference when comparing two contrasts. A larger reduction in absolute interocular difference from high to low contrast was related to a greater enhancement in binocular summation. This is to be expected given the established link between interocular difference and binocular summation.

Decrease in binocular summation has been found to be associated with increasing age (Pardhan, 1996, 1997; Pineles et al., 2011) especially at low contrast (Gagnon and Kline, 2003; Pineles et al., 2011). The neural noise hypothesis (Campbell and Green, 1965) and age-related neural and vision changes have been put forward as a potential explanation for this observed relationship (Gagnon and Kline, 2003). According to the hypothesis, summing of signal and uncorrelated noise between the two eyes leads to neural summation for contrast. The decline in binocular summation with age can be possibly accounted for by increased noise (Gagnon and Kline, 2003), neuronal cell loss (Weale, 1982), increased neural variability (Elliott et al., 1989; Whitaker et al., 1992) and/or larger interocular difference (Pineles et al., 2014). Although all observers in the current study are young adults, our data nonetheless show decline in binocular summation with advancing age for low contrast visual acuity. Furthermore, our findings indicate that when comparing low to high contrast, the change of binocular summation decreases, even to a negative value (i.e., less binocular summation at low contrast than at high contrast), as age increases. It is anticipated that the similar age effect could be observed in elderly individuals with normal vision. Given the common age-related ocular deteriorations such as cataract (Klein et al., 1992) and decline in scotopic and photopic sensitivity (Jackson and Owsley, 2000), the effect size may differ.

Bailey-Lovie letter charts adopted ten British letters with similar legibility as testing stimuli and was carefully designed to ensure little variation in the average difficulty between rows (Bailey and Lovie-Kitchin, 2013). However, as observer’s letter recognition deteriorated, there was within-row variation in recognition accuracy (i.e., the proportion of correct responses varied across letters in a row). It may be attributable to the combination of crowding effect and variation in letter confusability. In Bailey-Lovie letter charts (Bailey and Lovie-Kitchin, 2013), the spacing between neighboring letters was equal to the width of each letter in order to control (but not eliminate) potential contour interaction and crowding effect (Flom et al., 1963; Flom, 1991). Crowding effect remained evident near the acuity limit. Considering only the letters within a row, the first and the last letters had flanking letters merely on one side (right or left) and therefore were considered less crowded than the middle letters that were flanked on both sides. For example, the first letters in the last two rows of the high contrast chart had higher recognition accuracy than the middle letters by a difference of up to about 40% (Figure 1). A similar trend was also apparent in the low contrast chart when recognition performance degraded near acuity limit. Crowding did not account for all within-row variation in letter recognition accuracy. For instance, the third row from the last in the low contrast chart contained letters Z, R, U, H, and D from left to right. While crowding was expected to be comparable for the middle three letters, recognition accuracy for letter U was consistently higher than the accuracies for letters R and H in both monocular and binocular measures. The variation of letter difficulty was possibly due to the difference in letter confusability (similarity/confusion among letters).

In summary, the present study used Bailey-Lovie charts to measure binocular acuity summations and evaluated relationships among various measurements. We identified a positive relationship in binocular acuity summation between high and low contrast, and a significant association between a baseline measure and the change in binocular summation between contrast levels. These findings can serve as a reference in clinical practice and research when high and low contrast binocular summations are measured in assessing binocular functional vision. Future research should investigate whether the similar relationships exist in elder people who experience normal age-related visual deterioration, and how the relationships may change for patients with various visual disorders.

## Data availability statement

The data supporting the conclusions of this article are available by request from the corresponding author.

## Ethics statement

The studies involving human participants were reviewed and approved by the Institutional Review Board of The Ohio State University. The patients/participants provided their written informed consent to participate in this study.

## Author contributions

DY contributed to the design, data collection and analyses, and manuscript writing and editing. EW contributed to the data collection and analyses, and manuscript editing. Both authors contributed to the article and approved the submitted version.
