# Supplementary material for: Binocular summation in high and low contrast letter acuities
Source: Front Neurosci. 2023 Jun 15;17:1174900. doi: 10.3389/fnins.2023.1174900 (PMC10309007; doi:10.3389/fnins.2023.1174900)
Supplement: Supplementary file 1 [file Presentation_1.pdf]

Appendix A

There were 156 normal vision observers (99 females and 57 males) aged 18 to 37 years. At both contrast levels, visual acuity was similar between the right and the left eye with the difference ranging between -0.18 and 0.08 LogMAR for high contrast (mean = -0.001 LogMAR; paired t-test: $t(155) = -0.33$, $p = 0.74$) and -0.22 and 0.18 LogMAR for low contrast (mean = 0.004 LogMAR; paired t-test: $t(155) = 0.67$, $p = 0.50$; Figure A2A). Binocular acuity was consistently better than monocular (right, left, better, and worse eye) acuities (ps < 0.001). Interocular difference was found in 71% of the observers for high contrast (a difference of -0.18 LogMAR or less) and 89% for low contrast (-0.22 LogMAR or less; Figure A2B). Among the observers who had interocular difference at both contrast levels, 34% switched their better eye when contrast level changed. Among all the observers, 81% showed some degree of binocular summation for high contrast chart (up to 0.14 LogMAR) and 90% for low contrast chart (up to 0.20 LogMAR; Figure A2C). The rest of the observers either had no change or showed a small amount of binocular inhibition.

Besides the correlations shown in Tables A1 and A2, we also found correlation between age and binocular summation for low contrast chart ($r = -0.25$, one-tailed $p = 0.001$). No correlation was found between age and interocular difference. Binocular summation at low contrast correlated positively with summation at high contrast ($r = 0.19$, one-tailed $p = 0.01$). The correlation between change of binocular summation (between the two contrast levels) and baseline measurement was significant (Oldham's method: $r = 0.28$, $p < 0.001$). The change of binocular summation was also found to correlate negatively with the change of absolute interocular difference ($r = -0.23$, $p = 0.005$) and age ($r = -0.23$, $p = 0.005$).

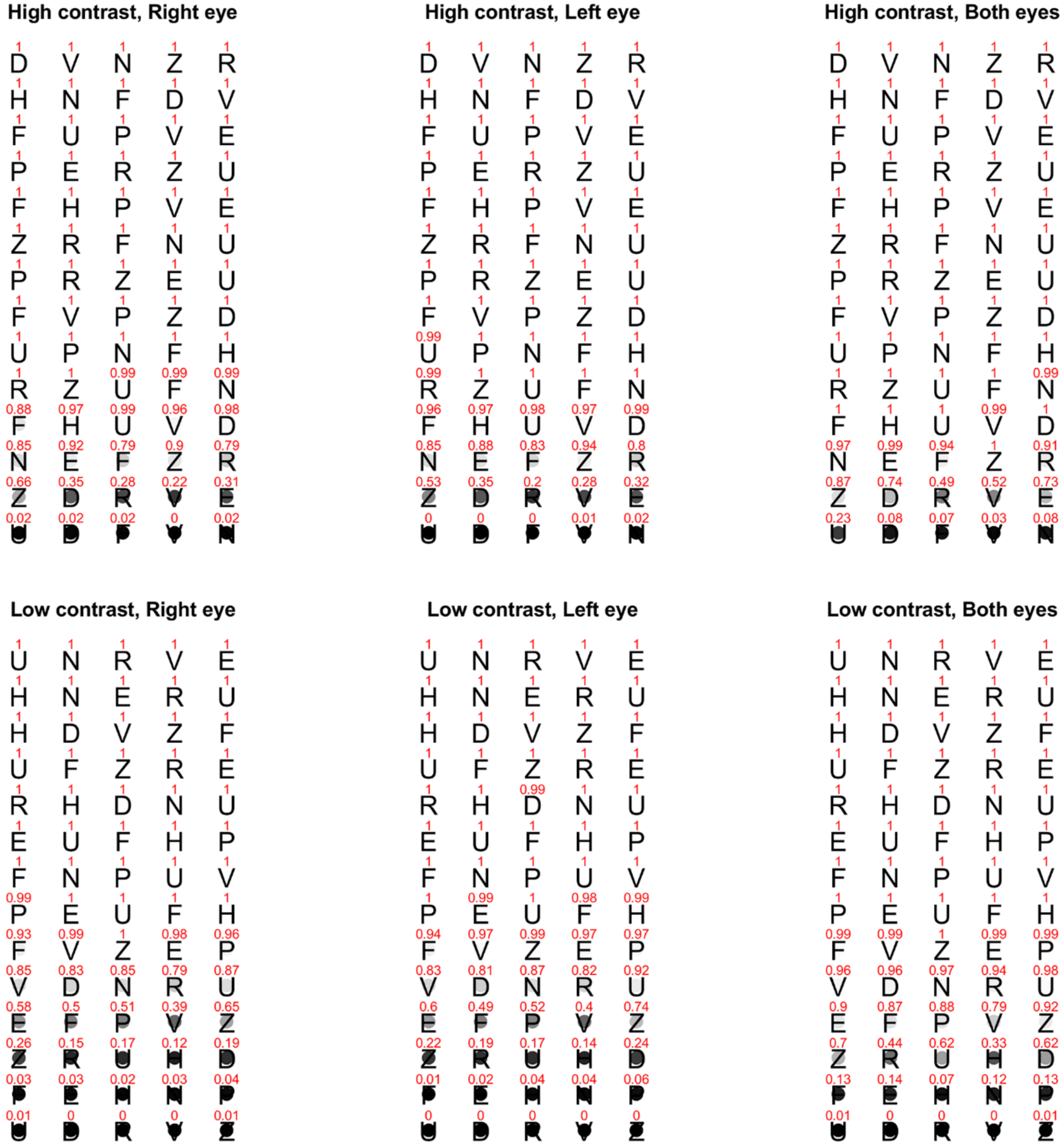

Figure A1. Proportion of correct responses for each letter on the high and low contrast Bailey-Lovie charts that were used for measuring monocular and binocular visual acuities at three-meter viewing distance.

	**High Contrast**	**Low Contrast**	**High - Low**	***r***	**one-tailed *p***
**Monocular (Right)**	-0.018±0.004	0.146±0.008	-0.164±0.006	0.52	< 0.001
**Monocular (Left)**	-0.017±0.004	0.142±0.008	-0.159±0.006	0.55	< 0.001
**Monocular (Better)**	-0.033±0.003	0.117±0.007	-0.151±0.006	0.55	< 0.001
**Monocular (Worse)**	-0.002±0.003	0.170±0.008	-0.172±0.006	0.54	< 0.001
**Binocular**	-0.072±0.003	0.051±0.006	-0.123±0.005	0.54	< 0.001
**Interocular difference**	-0.031±0.002	-0.053±0.004	0.022±0.004	0.29	< 0.001

**Binocular Summation**	0.039±0.003	0.066±0.004	-0.027±0.004	0.19	0.01

Table A1. Monocular and binocular acuities, interocular differences and binocular summations for the two contrast levels, the differences between the two contrast levels (Mean ± SE in LogMAR), and correlations between the two contrast levels.

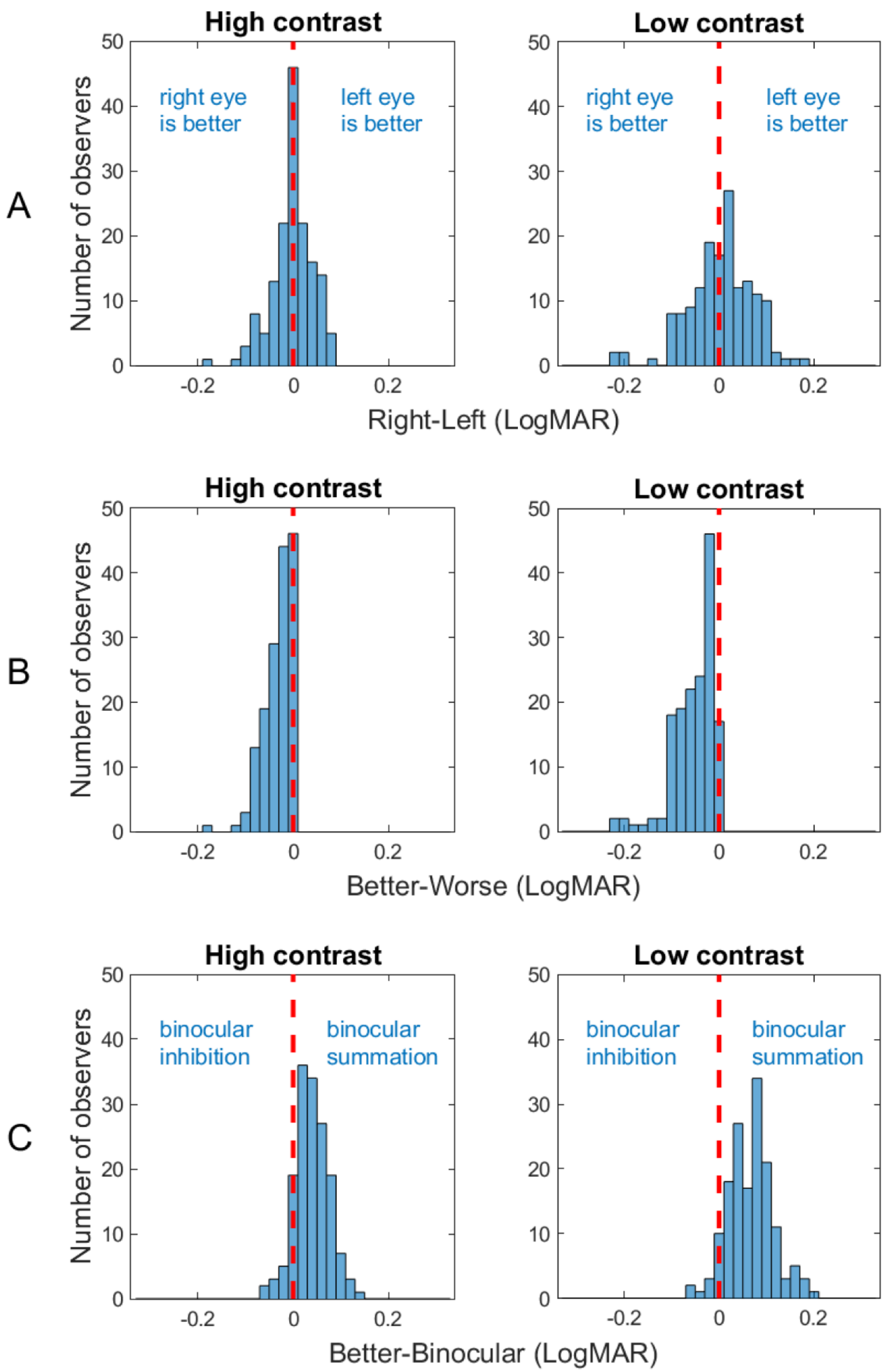
A
B
C
High contrast
Low contrast
Number of observers
right eye
is better
left eye
is better
Right-Left (LogMAR)
Better-Worse (LogMAR)
binocular
inhibition
binocular
summation
Better-Binocular (LogMAR)
50
40
30
20
10
0
-0.2
0
0.2

Figure A2. Frequency histograms of acuity difference between the right and the left eye (A), interocular difference (B), and binocular summation (C) for high and low contrasts.

		High Contrast		Low Contrast	
		r	one-tailed *p*	*r*	one-tailed *p*
**Binocular**	Better	0.64	< 0.001	0.84	< 0.001
	Worse	0.56	< 0.001	0.80	< 0.001
**Binocular summation**	|Interocular difference|	-0.31	< 0.001	-0.20	0.007

Table A2. Correlations between binocular and monocular (better or worse) acuity, between binocular summation and absolute interocular difference for the two contrast levels.

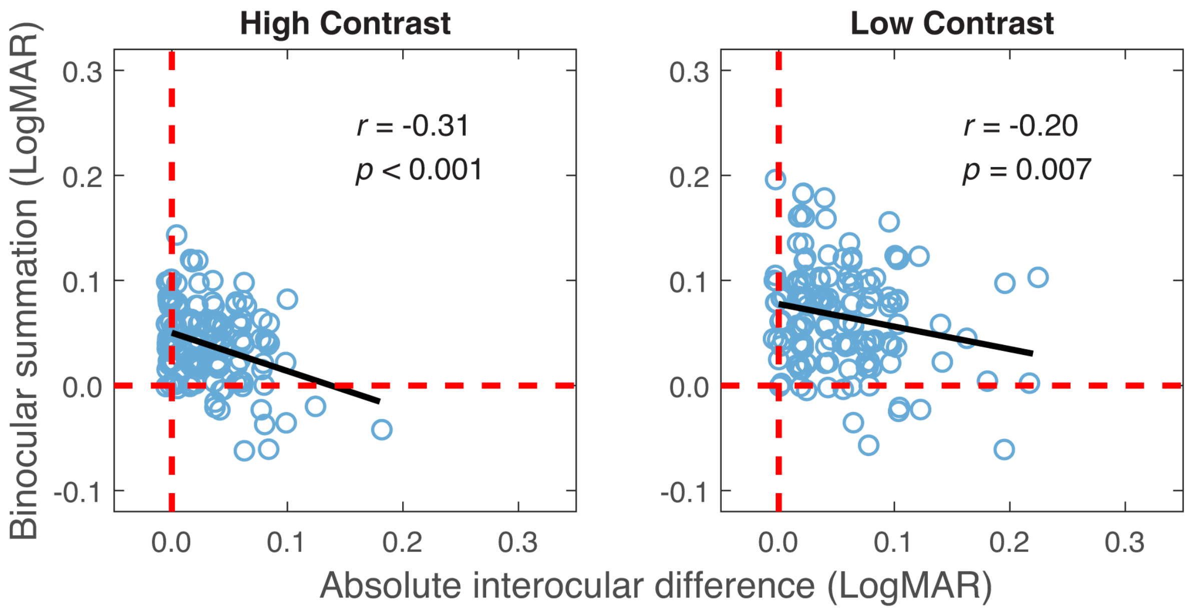

Figure A3. Binocular summation plotted against absolute interocular difference for high and low contrasts.

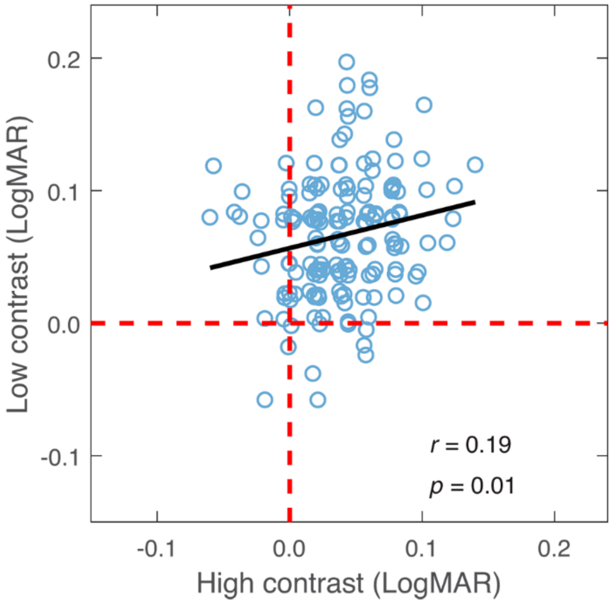

Figure A4. Binocular summation at low contrast plotted against binocular summation at high contrast.

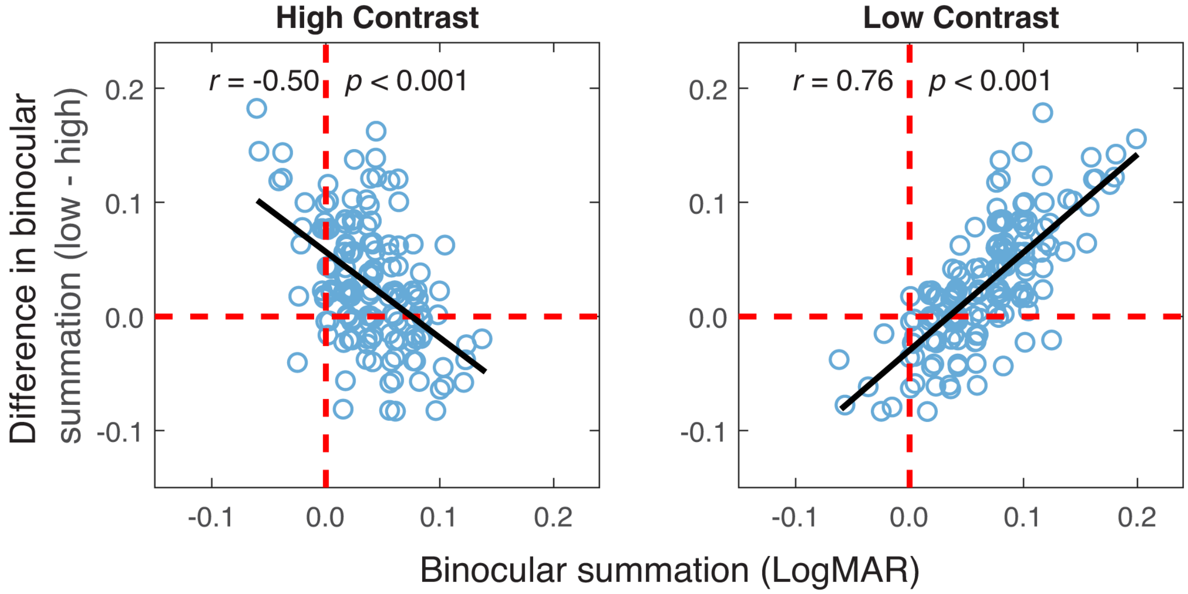

Figure A5. The difference in binocular summation between the two contrast levels plotted versus binocular summation at high and low contrast levels. Oldham's method is used to confirm that the effect of contrast depends on baseline.

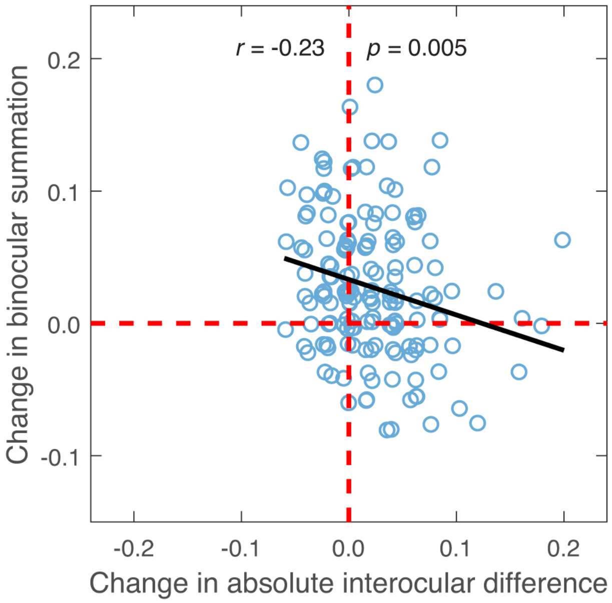

Figure A6. The difference in binocular summation plotted against the difference in absolute interocular difference (LogMAR).
